# Sedimentation Velocity Analytical Ultracentrifugation of Oxidized Recombinant Full-Length Factor VIII

**DOI:** 10.3389/fimmu.2020.00150

**Published:** 2020-02-07

**Authors:** Philip M. Zakas, John F. Healey, Ian W. Smith, David Lillicrap, Pete Lollar

**Affiliations:** ^1^Department of Pathology and Molecular Medicine, Queen's University, Kingston, ON, Canada; ^2^Department of Pediatrics, Aflac Cancer and Blood Disorders Center, Children's Healthcare of Atlanta, Emory University, Atlanta, GA, United States

**Keywords:** hemophilia A, factor VIII, oxidation, immunogenicity, analytical ultracentrifugation

## Abstract

Anti-drug antibodies to coagulation factor VIII (fVIII), often termed inhibitors, present the greatest economical and treatment related obstacle in the management of hemophilia A. Although several genetic and environmental risk factors associated with inhibitor development have been identified, the precise mechanisms responsible for the immune response to exogenous fVIII therapies remain undefined. Clinical trials suggest there is an increased immunogenic potential of recombinant fVIII compared to plasma-derived products. Additional biochemical and immunological studies have demonstrated that changes in recombinant fVIII production and formulation can alter fVIII structure and immunogenicity. Recently, one study demonstrated increased immunogenicity of the recombinant fVIII product Helixate in hemophilia A mice following oxidation with hypochlorite (ClO^−^). It is widely reported that protein aggregates within drug products can induce adverse immune reactions in patients. Several studies have therefore investigated the prevalence of molecular aggregates in commercial recombinant products with and without use-relevant stress and agitation. To investigate the potential link between oxidation-induced immunogenicity and molecular aggregation, we analyzed the recombinant fVIII product, Helixate, via sedimentation velocity analytical ultracentrifugation following oxidation with ClO^−^. At 80 μM ClO^−^, a concentration that reduced the specific-activity by 67%, no detectable increase in large molecular aggregates (s > 12 S) was observed when compared to non-oxidized fVIII. This lack of aggregates was demonstrated both in commercial excipient as well as a HEPES buffered saline formulation. These data suggest that oxidation induced immunogenicity is independent of aggregate-mediated immune response. Therefore, our data support multiple, independent mechanisms underlying fVIII immunogenicity.

## Introduction

The standard of care for patients with hemophilia A is prophylactic treatment with concentrated exogenous factor VIII (fVIII) products to prevent and control bleeding and mitigate joint damage. Commercial fVIII products, whether plasma-derived or recombinant, result in a significant incidence of anti-drug antibodies (ADAs), termed inhibitors, which prevent fVIII activation (1), interfere with co-factor function to factor IX (1), abrogate phospholipid and/or platelet binding (2), or enhance drug clearance (3). These inhibitors can occur in upwards of 40% of previously untreated patients (4–6) and recent clinical studies have suggested that recombinant products may be more immunogenetic (7–9). Current theories for immune complex formation within recombinant products include (i) the absence of the immuno-protective effect of von Willebrand factor, (ii) altered glycosylation patterns and structures due to various heterologous expression systems, and (iii) increased molecular aggregation of recombinant products prior to infusion. While the precise pathogenic mechanisms responsible for fVIII inhibitor development remain unclear, a multifactorial combination of genetic and non-genetic risk factors are suggested to influence the anti-fVIII immune response (10–13).

Non-genetic factors, such as inflammation, hemarthrosis, reactive oxygen species (ROS), and redox states are postulated to stimulate the anti-fVIII immune response. Hemarthrosis prior to and during factor infusion was associated with an increased inhibitor response in rats with severe hemophilia A (14), suggesting that on-demand treatment may correlate with increased risk. Additionally, sites of endothelial damage during hemarthrosis precede the release of ROS species (15) which influence the local redox milieu. A recent investigation of the effect of oxidation demonstrated an increased immune response in hemophilia A mice when fVIII was oxidized with ClO^−^ prior to administration (16). ClO^−^ oxidation of fVIII compromised its procoagulant activity but did not abrogate VWF binding, demonstrating that oxidation alters the fVIII structure outside of the VWF binding sites and that VWF did not protect from an immune response to the oxidized fVIII species. The concentration of coagulation factors at sites of injury and inflammation may therefore result in undesirable structural changes which alter the immunogenicity of the protein.

Oxidation is widely reported to increase the immunogenicity of proteins including interferon alpha2b (17), collagen type II (18), and ovalbumin (19). A link between oxidative induced aggregate formation and subsequent immunogenicity has been demonstrated with human interferon-β (20). Protein aggregates in commercial products, as a result of altered structure and assemblies, also increase the risk of ADAs in patients (21, 22). These altered assemblies can result from chemical reactions as previously described, or result from accumulated misfolded protein. Recombinant fVIII is produced in heterologous expressions systems and it has been reported that 2nd generation products Helixate and Kogenate are more immunogenic than the 3rd generation product Advate (23, 24). It has also been demonstrated that these products contain significantly higher levels of aggregates before and after use-related stress as measured by size exclusion high-performance liquid chromatography, dynamic light scattering, and sedimentation velocity analytical ultracentrifugation (SV-AUC) (25–27). In the present study, we use SV-AUC to investigate the effect of oxidation of recombinant fVIII product Helixate on the formation of molecular aggregates.

## Methods

### Materials

Sodium hypochlorite (NaOCl) was purchased from Sigma-Aldrich (St. Louis, MO). NaOCl concentration was determined using an extinction coefficient of 350 M^−1^ cm^−1^ at 292 nm in water. Zeba Spin desalting columns and Amicon Ultra centrifugal filters were purchased from Thermo Fisher Scientific (Waltham, MA) and Millipore Sigma (Burlington, MA), respectively. Helixate FS (CSL Behring, Kankakee, IL, USA; Lots 270PP4J and 27N1VK1) was purchased from the manufacturer and reconstituted using sterile water in accordance with the kits and instructions provided.

### Factor VIII Preparation

For some experiments, Helixate was exchanged into 0.15 M NaCl, 0.02 M HEPES, 5 mM CaCl_2_, 0.01% polysorbate 80 (w/w), pH 7.4 (HBS/Ca/PS-80) with a Zeba Spin desalting column or by repeated filtration using Amicon Ultra-15 Ultracel-30K centrifugal filter, which was passivated with (HBS/Ca/PS-80) prior to buffer exchange. Helixate was oxidized by addition of sodium hypochlorite for ~10 min followed by buffer exchange.

Specific-activity of Helixate was determined by one-stage coagulation assay using a Diagnostica Stago Start (Parsippany, NJ) viscosity-based hemostasis analyzer and referenced to pooled citrated normal plasma (FACT). Activity was normalized to A_280_ mass determination following extinction coefficient corrections using an extinction coefficient of 1.2 (mg/mL)^−1^ cm^−1^ based on tyrosine, tryptophan and cysteine composition and molar mass. Specific-activities were measured immediately following addition of ClO^−^ and remained stable over 24 h. fVIII deficient plasma and FACT reference were purchased from George King Biomedical (Overland Park, KS). Automated APTT reagent was purchased from Trinity Biotech (Wicklow, Ireland).

### Analytical Ultracentrifugation

SV experiments were performed at 105,000 *g* (38,000 rpm) at 20°C in a Beckman Coulter ProteomeLab XLI analytical ultracentrifuge. Scanning was done at 280 nm in an An-60 rotor equipped with 12 mm pathlength double sector cells and sapphire windows. Sample and reference buffer volumes were 0.40 mL each. Scans were initiated in continuous mode at ~4 min intervals using a radial spacing of 0.003 cm after reaching the target rotor speed and were acquired at ~3 min intervals.

Data were analyzed using the continuous c(s) distribution model in SEDFIT, version 16.1c (28, 29), or the hybrid local continuous/global discrete species model in SEDPHAT, version 15.2b (http://analyticalultracentrifugation.com). These models produce a least squares fit of the absorbance signal as a function of radial position and time to a set of Lamm equations corresponding to a user-defined range and increments of sedimentation coefficients, yielding a sedimentation coefficient distribution, c(s), and a signal-average frictional ratios, f/f_o_. The simplex algorithm and Marquardt-Levenberg algorithms were used for both programs. The meniscus position, baseline, and time-invariant noise also were fitted. Continuous c(s) distribution fitting was done using maximum entropy regularization with a confidence interval of 0.68. Hybrid local continuous/global discrete species modeling was done using Tikhonov regularization with a confidence interval of 0.68.

The molar mass, M, of the dominant species in formulated Helixate was estimated using

$$\begin{array}{l} M=\;9\pi \sqrt{2\;}\;N_{a}{\bar{\nu }}^{1/2}{\left(\frac{s\;\eta \frac{f}{f_{0}}}{\left(1-\bar{\nu }\rho \right)}\right)}^{\frac{3}{2}} \end{array}$$

where η is the solvent viscosity, ρ is the solvent density, $\bar{\nu }$ is the partial specific volume of fVIII, and *N*_*a*_ is Avogadro's number. A value of 0.719 mL/g was used for the partial specific volume of fVIII (25).

The sedimentation coefficients were converted to the standard condition of water as reference solvent at 20°C ((s_w_)_20,w_) as described by Svedberg and Pedersen (30). The viscosity and density of the formulation buffer and HBS/Ca/Tw were measured in a Lovis 2000M viscometer and an Anton Paar DMA4500 densitometer. SV graphs were plotted using GUSSI version 1.4.2 (31).

## Results

### Oxidative Inactivation of fVIII

**Figure 1:**
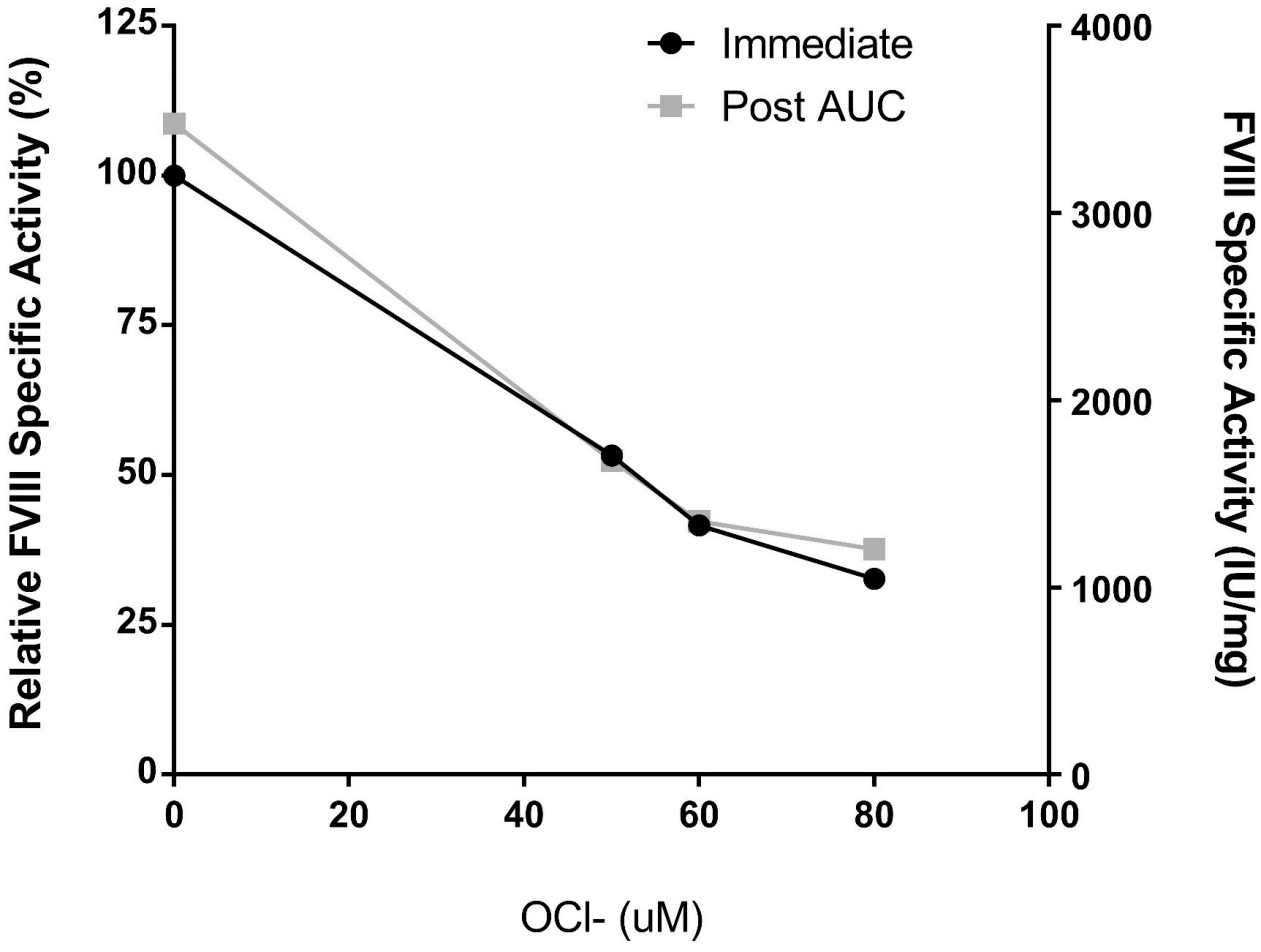
Helixate specific-activity reduction following oxidation. Helixate was reconstituted in ½ volume of Sterile Water for Injection resulting in 2x formulation buffer. Following oxidation with molar excess ClO^−^, Helixate was exchanged into HBS/Ca/PS-80 and specific-activity was determined (black circles). FVIII specific-activity was measured by one-stage coagulation activity and plotted as specific activity relative to the absence of ClO- as well as absolute specific activity. Specific-activity of oxidized Helixate was also measured following completion of AUC (gray squares) to confirm no loss of activity during AUC.

Peyron et al. recently demonstrated that oxidation of Helixate reduced procoagulant function and increased immunogenicity without affecting binding to VWF (16). While the mechanism of the observed immunity is not yet understood, oxidation is suggested to alter the structure and/or assembly of the protein. To analyze the formation of protein aggregates in the fVIII product, Helixate, by SV AUC, dose-finding studies were conducted to determine the concentration of ClO^−^ required to abrogate procoagulant function and therefore alter the physical structure of fVIII. To ensure that active and/or inactive fVIII mass was not removed following buffer exchange, A_280_ measurements were taken before and after oxidation and specific-activity (IU/mg) is reported. Exposure of fVIII to concentrations of 50, 60, and 80 μM ClO^−^ in 2x formulation buffer for 10 min prior to buffer exchange to remove ClO^−^ resulted in 47, 58, and 67% reduction in specific-activity (Figure 1). These concentrations represent a 55–88-fold molar excess of ClO^−^ over fVIII. The concentrations required to reduce Helixate specific activity in 2x formulation buffer were roughly double those required in HBS/Ca/PS-80 (data not shown), likely due to the presence of the anti-oxidant histidine (Table 1). As a control against subsequent structural changes and loss of coagulant activity because of the 8 h SV AUC process, specific-activity of samples were tested immediately following centrifugation. Samples demonstrated no change in specific activity 8 h after initial testing (Figure 1).

**Table 1:** Stabilizers and excipients in helixate.

**Stabilizers**	**1x**	**2x**
Sucrose	0.9–1.3%	1.8–2.6%
Glycine	21–25 mg/mL	42–50 mg/mL
Histidine	18–23 mg/mL	36–46 mg/mL
**Inactive ingredients/excipients**		
Sodium	27–36 mEq/L	54–72 mEq/L
Calcium	2.0–3.0 mEq/L	4.0–6.0 mEq/L
Chloride	32–40 mEq/L	64–80 mEq/L
Polysorbate 80	64–96 μg/mL	128–192 μg/mL
Sucrose	28 mg/vial	56 mg/vial

### SV AUC of Oxidized fVIII

**Figure 2:**
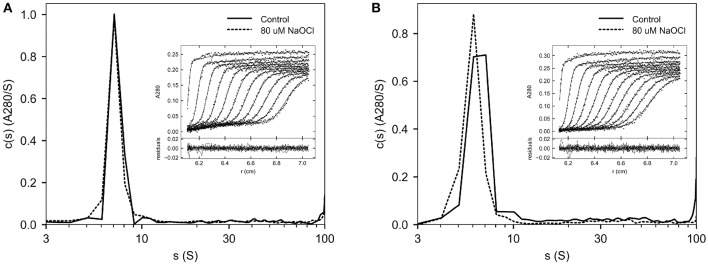
SV AUC of oxidized Helixate. Helixate in HBS/Ca/PS-80 **(A)** or 2x formulation buffer **(B)** was treated with either 80 μM ClO^−^ (dashed curve) or control buffer (solid curve). Following buffer exchange using a desalting column, samples were subjected to SV AUC at 105,000 *g* at 20°C. A_280_ scans were fitted to a continuous c(s) distribution from 0 to 100 S at 1 S increments. The insets show the fitted data for control samples. Only every fourth scan and every other data point are shown for clarity. The lower panels in the insets show the residuals of the fitted data.

To assess the formation of aggregates following oxidation, Helixate was desalted into HBS/Ca/PS-80, and exposed to 80 μM ClO^−^ or control buffer for 10 min. Helixate then underwent buffer exchange into HBS/Ca/PS-80 to remove ClO^−^ and was subjected to SV AUC. Figure 2A shows an overlay of the 80 μM ClO^−^ and control Helixate sedimentation coefficient distributions. Diffuse aggregates are evident in the region between 12 and 100 S in the control sample as observed previously for Helixate (25). The extent of aggregation was similar in the oxidized sample. Aggregates in the 12–100 S represented 13.4 and 13.3% of the signal relative to the region between 5 and 100 S for the control and 80 μM samples, respectively.

For therapeutic use, Helixate is reconstituted with sterile water for injection, which produces a solution with excipient and stabilizer concentrations listed in Table 1. To evaluate the oxidation of Helixate in the excipient/stabilizer buffer system, and to increase the 280 nm absorbance signal for SV AUC for increased potential of detecting a differential increase in aggregates due to oxidation, Helixate was reconstituted at twice the concentration of the therapeutic formulation and exposed to control buffer or 80 μM ClO^−^. Figure 2B shows that aggregation was similar between oxidized and control fVIII. Aggregates in the 12–100 S region were 14.5 and 9.2% relative to the region between 5 and 100 S for the control and 80 μM samples, respectively.

**Figure 3:**
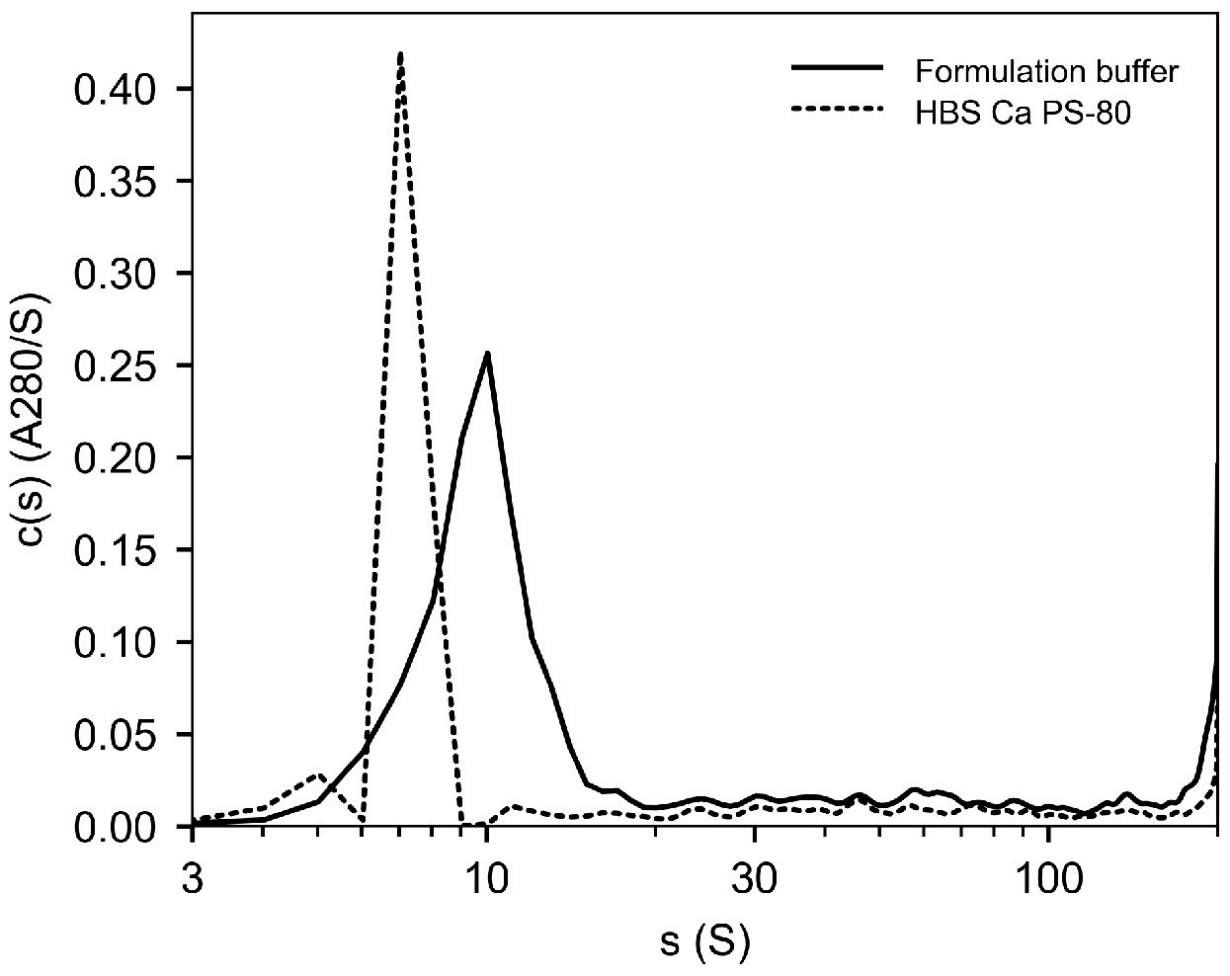
SV AUC of Helixate in formulation buffer and HBS/Ca/PS-80. Helixate was reconstituted in Sterile Water for Injection to produce its therapeutic formulation (solid curve) or exchanged into HBS/Ca/PS 80 (dashed curve) and immediately subjected to SV AUC at 105,000 g at 20°C. A_280_ scans were fitted to a continuous c(s) distribution from 0 to 200 S.

As an additional control, we determined whether aggregates were removed from Helixate during buffer exchange procedure. This potentially would produce a false negative result in which oxidized fVIII aggregates were selectively removed by a solid phase matrix. Figure 3 shows the c(s) distributions of Helixate in formulation buffer and following desalting into HBS/Ca/PS-80, revealing a major peak with an (Sw)20, *w*-value of 7.6 S. Aggregate levels were 18.9% and 15.9% in formulation buffer and HBS/Ca/PS-80, respectively, indicating that aggregates do not become trapped on the solid phase matrix. To further confirm this, SV AUC was performed on oxidized Helixate without removal of HOCl, and no increase in diffuse aggregates was observed (data not shown).

The integration range for Helixate in formulation buffer was 15–100 S instead of 12–100 S because dominant species in Helixate sediments faster in formulation buffer than in HBS/Ca/PS-80 (Figure 3). This is surprising because the nominal excipients and stabilizers in Helixate produce a ~0.3 M glycine/0.03 M sucrose solution. Therefore, the predicted density and viscosity of formulation buffer would be greater than HBS/Ca/PS-80 and thus slow the sedimentation of fVIII. To investigate this phenomenon, the density and viscosity of Helixate in formulation buffer was measured and SV AUC was performed on the same sample. The density and viscosity of the formulation buffer were 1.0147 g/mL and 0.01095 Poise, respectively, compared to 1.0062 g/mL and 0.01028 Poise of HBS/Ca/PS-80.

**Figure 4:**
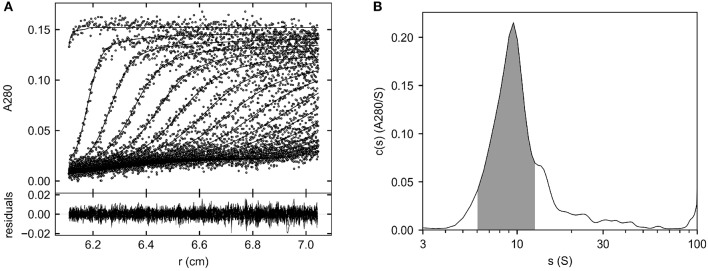
Major species of Helixate in formulation buffer determined by SV AUC. Helixate was reconstituted in Sterile Water for Injection to produce its therapeutic formulation. The density and viscosity of the formulated solution were measured as described in Methods and the sample was subjected to SV AUC at 105,000 g at 20°C. **(A)** Fitted absorbance scans. Only every fourth scan and every other data point are shown for clarity. Lower panel, residuals of the fitted data. **(B)** Continuous c(s) distribution from 0 to 100 S. The shaded area corresponds to the region used to determine the signal average sedimentation coefficient of the dominant species.

Figure 4 shows the fitted sedimentation profiles and c(s) distribution of Helixate in formulation buffer. The shaded region in Figure 4B was integrated, producing a signal-average sedimentation coefficient, s_w_, of 9.14 S. This corresponds to a value at 20°C in water, (s_w_)_20,w_, of 10.43 S. In contrast, the (s_w_)_20,w_ of the fVIII heterodimer in Helixate is 7.6 S in HBS/Ca/PS-80 (Figure 3). Continuous c(s) distribution analysis, which produces an estimate of the signal-average frictional ratio, f/f_o_, of the entire sedimenting population (29), yielded a value of 1.29. To exclude the possibility that aggregates were significantly contributing to the frictional ratio, the hybrid local continuous/global discrete species model in SEDPHAT, which provides an estimate of the frictional ratio in user-defined regions within the sedimentation coefficient distribution range of interest (32), was implemented and produced an estimated frictional ratio of 1.28. In contrast, the frictional ratio of the fVIII heterodimer in HBS/Ca/PS-80 is 1.91. The frictional ratio is a measure of the departure of the sedimenting particle from spherical symmetry and is inversely related to the sedimentation coefficient. The sedimentation coefficient and frictional ratio, combined with the solvent density and viscosity and partial specific volume of the protein, produced an estimated signal-average molar mass 213,000 g/mol for molecule(s) corresponding to the shaded region in Figure 4B as described in Methods. This is close to the mass estimated for the fVIII heterodimer in HBS/Ca/PS-80 (25). These results are consistent with the dominant species in formulated Helixate being the fVIII heterodimer that sediments faster in formulation buffer due to a dramatic decrease in the frictional ratio. The frictional ratio of fVIII in formulation buffer is typical of a globular protein whereas the value in HBS/Ca/PS-80 of 1.9 is due to a significant departure from spherical symmetry. Scanning transmission electron microscopy studies indicate that the B domain projects from the body of porcine fVIII as a long stalk (33), which could explain the relatively large frictional ratio of the fVIII heterodimer. Conceivably, the B domain packs next to the body of fVIII in HBS/Ca/PS-80 buffer, reducing its frictional ratio.

## Discussion

This study demonstrates that oxidation of Helixate, although damaging to procoagulant function, does not result in increased large molecular weight aggregates. SV AUC analysis following oxidation in a HEPES buffer or manufacturer's formulation buffer revealed an equivalent proportion of aggregates compared to non-oxidized Helixate, which was similar to previous reports (25). Buffer exchange using a desalting column did not remove aggregates from Helixate and the total A_280_ was unchanged before and after oxidation. The immunogenicity of fVIII is independent of its procoagulant function (34, 35) but is, in part, regulated by association with von Willebrand factor (VWF) (34, 36). Oxidation of Helixate with ClO^−^ was previously shown not to affect VWF binding (16). Therefore, the mechanism by which oxidized fVIII induces an increased immune response remains unanswered, however, it is not protected by VWF binding. Aggregates within protein biologics, including fVIII (37), are known to increase the immunogenic potential of the protein drug. It is conceivable that the structural and chemical changes caused by oxidation of Helixate result in increased or novel epitope exposure or altered antigen recognition following administration. Modifications to the protein which are not detected as changes in sedimentation rate, size, or frictional ratio must be occurring due to the loss of procoagulant activity, and these deformations must be further investigate for a better understanding of fVIII immunogenicity. Taken together, this study supports multiple independent mechanisms of immunogenicity that contribute to the complexity of ADA formation.

Herein we highlight the importance of formulation composition for biologic drugs with heterogeneous populations, such as fVIII. Within marketed recombinant fVIII products, buffers, and stabilizers have changed significantly across product generations without sufficient analysis into their propensity to aggregate, until recently (26). In this study, we demonstrate a significant difference in fVIII conformation in HBS/Ca/PS80 buffer compared to 1x or 2x formulation buffer, measured by frictional ratio (Figure 3). This resulted in the dominant species of fVIII in formulation buffer to distribute over a larger c(s) range (Figure 4). In light of recent studies suggesting increased immunogenicity in recombinant products compared to plasma derived (38, 39), the stability and uniformity of recombinant fVIII products prior to administration is of great importance. One such complication is the inconsistent concentration of excipient stabilizers across lots of a single recombinant product, normalized to fVIII IU/ml (25). Excess or deficient solute within the formulation buffer alters the density, viscosity, and frictional ratio which therefore alters the homogeneity of the fVIII product. Furthermore, absence of sufficient redox protectants can increase the propensity to form ADA following administration. To this effect, we also measured fVIII activity in NaHPO_4_ buffer following OCl-oxidation (data not shown). In this buffer devoid of alternative oxidation targets such as HEPES, PS-80, histidine, glycine, etc., the IC50 was determined at only 5-fold molar excess compared to 55-fold in 2x formulation buffer. Therefore, while the mechanism of oxidation-induced immunity is not driven by aggregate formation and remains unexplained, the inclusion of improved buffers, stabilizers, and antioxidants may contribute to a reduced incidence of anti-fVIII inhibitors.

## Data Availability Statement

All datasets for this study are included in the article/supplementary material.

## Author Contributions

PZ, IS, JH, and PL planned and performed experiments, interpreted data, and participated in writing the paper. DL interpreted data and participated in writing the paper.

### Conflict of Interest

PL is inventor on patents owned by Emory University claiming compositions of matter that include modified FVIII proteins with reduced reactivity with anti-FVIII antibodies. DL receives grant support from Bayer, Bioverativ, CSL, and Octapharma. The remaining authors declare that the research was conducted in the absence of any commercial or financial relationships that could be construed as a potential conflict of interest.
